# Swellix: a computational tool to explore RNA conformational space

**DOI:** 10.1186/s12859-017-1910-7

**Published:** 2017-11-21

**Authors:** Nathan Sloat, Jui-Wen Liu, Susan J. Schroeder

**Affiliations:** 101 Stephenson Parkway, Norman, OK 73019 USA

**Keywords:** RNA structure prediction, RNA ensembles, Conformational space, RNA motif search

## Abstract

**Background:**

The sequence of nucleotides in an RNA determines the possible base pairs for an RNA fold and thus also determines the overall shape and function of an RNA. The Swellix program presented here combines a helix abstraction with a combinatorial approach to the RNA folding problem in order to compute all possible non-pseudoknotted RNA structures for RNA sequences. The Swellix program builds on the Crumple program and can include experimental constraints on global RNA structures such as the minimum number and lengths of helices from crystallography, cryoelectron microscopy, or in vivo crosslinking and chemical probing methods.

**Results:**

The conceptual advance in Swellix is to count helices and generate all possible combinations of helices rather than counting and combining base pairs. Swellix bundles similar helices and includes improvements in memory use and efficient parallelization. Biological applications of Swellix are demonstrated by computing the reduction in conformational space and entropy due to naturally modified nucleotides in tRNA sequences and by motif searches in Human Endogenous Retroviral (HERV) RNA sequences. The Swellix motif search reveals occurrences of protein and drug binding motifs in the HERV RNA ensemble that do not occur in minimum free energy or centroid predicted structures.

**Conclusions:**

Swellix presents significant improvements over Crumple in terms of efficiency and memory use. The efficient parallelization of Swellix enables the computation of sequences as long as 418 nucleotides with sufficient experimental constraints. Thus, Swellix provides a practical alternative to free energy minimization tools when multiple structures, kinetically determined structures, or complex RNA-RNA and RNA-protein interactions are present in an RNA folding problem.

**Electronic supplementary material:**

The online version of this article (10.1186/s12859-017-1910-7) contains supplementary material, which is available to authorized users.

## Background

Approximately 80% of the human genome is transcribed into an RNA sequence, although only 2% of the genome codes for proteins [1]. This discovery reveals the abundance of noncoding RNA with as yet undetermined function. The flood of RNA sequence information from next generation high-throughput sequencing technology and the explosion of discoveries for non-coding RNA create an enormous need for RNA structure prediction tools. RNA structure prediction methods facilitate interpretation of sequence data to inform biological structure and generate testable hypotheses for function. RNA structure prediction tools form a key component in many genome-wide RNA analysis pipelines [2–5]. Many of these new RNA discoveries reveal RNA sequences with multiple functional folds or partially unfolded RNA [2, 4, 6, 7]. For example, one study estimates that 20% of RNA in human cells have multiple folds based on the existence of conflicting pairing constraints measured by in vivo crosslinking [2]. Thus, there is a need for tools that efficiently and thoroughly explore the conformational landscape of an RNA sequence. This paper presents a new computational method, Swellix, that computes efficiently all possible non-pseudoknotted structures for an RNA sequence by counting helices rather than base pairs. Swellix also counts RNA motif frequency, and thus provides insight into possible functional interactions that may not be present in low-energy structure predictions.

**Fig. 1:**
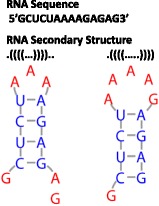
RNA Folding Problem. The sequence is the ordered list of A, C, G, and U nucleotides. The secondary structure is the set of Watson-Crick CG and AU base pairs. GU pairs may also be included. The RNA folding problem consists of how to best predict secondary structure (and ultimately three-dimensional structure and function) from sequence. For example, the sequence of a 14mer RNA oligonucleotide can fold into 119 secondary structures [21]. Two different hairpin secondary structures are shown graphically and in dot-parentheses notation. In dot-parentheses notation, dots represent unpaired nucleotides, and parentheses represent paired nucleotides. In the graph, unpaired nucleotides are red and paired nucleotides are blue. Vertical gray lines connect nucleotides in sequence and horizontal gray lines connect nucleotides in base pairs

The RNA folding problem is defined by base pairing rules for Watson-Crick and GU pairs. An RNA secondary structure consists of a set of base pairs, noncanonical pairs, and non-paired nucleotides (Fig. 1). A minimum number of 3 nucleotides is required for an RNA to fold back on itself and form a new helix. Each nucleotide may pair only once, and the pairs are nested, ie pseudoknots that cross previous pairs are not directly allowed. Thus, the RNA folding problem can be viewed as a maximum pair matching problem with complexity of O(N^3^), where N is the number of nucleotides. The number of possible structures is approximately 1.8^N^ [8]. The Nussinov algorithm approaches the RNA folding problem by finding the maximum scoring structure, or set of base paired nucleotides [9, 10]. The most common scoring function is based on thermodynamic parameters [11, 12] and free energy minimization [13]. The thermodynamic database is continually being updated and expanded [11, 12, 14]. The free energy minimization approach assumes that the lowest free energy structure is the most likely functional fold for the RNA sequence. This assumption, however, does not account for co-transcriptional RNA folding, kinetically determined RNA folds, potential RNA tertiary structure interactions, or RNA-protein interactions. Sampling suboptimal folds [15], computing base pair probabilities [16], and computing centroid structures [17] provides a broader view of the RNA conformational landscape than a single minimum free energy (MFE) structure but continue to use a thermodynamic-based scoring function. Additional experimental constraints can be combined with free-energy minimization in order to better predict functional RNA folds [12]. Recent advances focus on predicting RNA structures with 2 or 3 functional folds, but still rely on thermodynamic scoring functions [7].

The first approach to complete enumeration of RNA structures after the discovery of tRNA crystal structures took more than 3 days and led to the general misconception that it is not possible or practical to completely enumerate all the possible RNA secondary structures [18]. Utilizing free energy constraints to reduce the conformational space, the Wuchty algorithm computes a complete set of structures within a given energy window of the MFE structure [19, 20]. The Crumple algorithm used a different approach and modern supercomputing resources to compute all possible structures and then apply filters based on experimental data [21, 22]. Thermodynamic stability may be one criterion in the scoring function, but is not necessarily part of the scoring function. Crumple can include data from pairing constraints in phylogenetic analysis, SELEX experiments, or chemical and enzymatic probing experiments, as well as thermodynamic parameters. Crumple and the Sliding Windows and Assembly application of Crumple can incorporate constraints on the minimum number and length of helices from crystallography or cryoelectron microscopy data. Pairing constraints are the most powerful for reducing conformational space [21]. Crumple and even efficient parallelization of Crumple were limited by sequence length and long run times, however. The new Swellix program builds on the Crumple algorithm and is now able to compute all possible non-pseudoknotted structures for RNA sequences up to 418 nucleotides with sufficient helix constraints within 2 days with an XE6 node of the Blue Waters supercomputer, thus making many functional noncoding RNAs accessible to thorough analysis of conformational space. For example, the average length is 435 nucleotides for the 5,391,569 RNA sequences in the RFam database 12.1 [23].

The main conceptual advance in Swellix is to combine all possible helices rather than combine all possible base pairs in the generation of RNA structures. This helix abstraction is further developed by bundling together similar helices that exist in the same region of RNA sequence. The use of abstract representations for RNA helices has previously been applied to free energy minimization approaches [24], but has not been applied previously to complete enumeration methods. In addition, improvements in memory use, computational efficiency, and effective parallelization strategies at several points in the algorithm further enable the Swellix algorithm to generate all possible non-pseudknotted folds for an RNA sequence and provide a count of functional RNA motif occurrence. This approach provides an alternative to standard RNA structure prediction methods when the assumptions of a free energy minimization approach may not hold true.

## Methods

### Swellix program

**Fig. 2:**
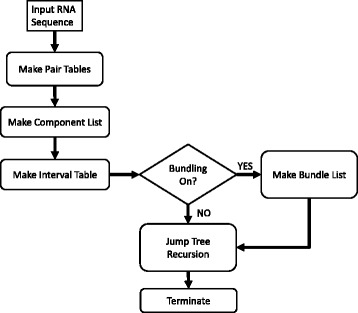
Flowchart of Swellix RNA Folding Program. The steps of the swellix program are shown in a flowchart. The bundling and make jump tree recursion were parallelized to improve run times

Swellix builds on the Crumple software [21, 25, 26]. Crumple focuses on individual base pairs and possible combinations thereof. Swellix considers helices as discrete components instead of base pairs. The Swellix algorithm is iteratively recursive and based on the core Crumple algorithm. However, there are multiple mechanisms that Swellix uses which differentiate the two algorithms. Swellix can be deconstructed into five major pieces (Fig. 2):

1. Constructing the Pair Table
2. Constructing the Component List
3. Constructing the Interval Look-up Table
4. Constructing the Bundle List
5. Iteratively Recursive Combinatorics (Make Jump Tree)

Parts one through four serve solely to speed up the recursive step. These are tables constructed to reduce the number of operations needed when checking if a valid helix exists within an interval. The bundling step serves to group similar structures together and reduce the size of the linked list that is sent to the recursive step. The iteratively recursive combinatorics step in Swellix is based on the algorithm in Crumple and has improvements in memory use and parallelization.

#### Constructing pair table

The input is an RNA sequence, or a string of A, C, G, and U that represent the 4 nucleotides in RNA. All pair-wise matching results of the entire RNA string are tabulated on a look-up table to speed up the extremely repetitive comparison procedure. The pairing rules are based on Watson-Crick (A-U and C-G) and wobble (G-U) base-pairing criteria. The data structure is a 2D integer array, where only the upper triangle region is used. The pseudo code is provided in supporting materials with time complexity O(n^2^), where n is the RNA sequence length.

#### Make component list

This phase features one of the key distinctions from the Crumple algorithm. Instead of iterating through the O(n^2^) process to identify the identical helices inside slightly different intervals *I*’s, in this phase, Swellix explores all qualified helices that will possibly be used in the entire runtime and documents them in an array of linked lists. Each array index *i* represents the nucleotide at the *i* ^*th*^ position of the input RNA sequence. Each node in the linked list extending from array cell *i* represents one component (ie, a helix) that begins at the *i* ^*th*^ nucleotide. In other words, the 5′-end outermost nucleotide of a helix is labeled the “type” of that component. Continuing with that terminology, the *i* ^*th*^ array cell represents the component type *i*, and all nodes of the linked list from the *i* ^*th*^ array cell share the same type (starting nucleotide).

The pseudo code is shown in the supporting materials, with the time complexity of O(Ln^2^) for n > > L, where L is the prescribed minimum helix length based on the experimental constraints. There are two points to making the component list. First, k_1 and k_2 indices, iterating through the entire RNA sequence, mark the boundary of a candidate helix. i, j are the helix pair indices making the k_3 stepwise matching checks starting from k_1 and k_2, respectively. Second, at the conditional statement ‘if(i, j) can be paired’, the pair table is called. The runtime saving for each if statement implementation is small, but the collective benefit is substantial.

#### Make interval look-up table

In the array of the component list, not all array cells (component types) have corresponding components, ie., some cells are empty. For some long RNA sequences with many experimental constraints, most of the array cells will be empty. To facilitate the Make Jump Tree process, it is worthwhile to tabulate the bounds for each interval so that the scanning process inside each interval may skip the empty array cells. The pseudo code is listed in supplemental material with the time complexity of O(mn^2^), where m is the number of components in the component list. The runtime saving of each empty-cell skip is tiny, but the collective benefit is substantial.

#### Make bundle list

In order to reduce the input for the Make Jump Tree phase and to facilitate identification of distinctly different structures in the output, a bundle of similar helices can be grouped together in one single representative structure. For example, if the following three substructures occurred in a span of 15 nucleotides,. ((((…..)))).,. ((.((….))))., and. ((..((…))))., then only one helix would be selected as the representative helix for the bundle, in this case. ((((……))))., the helix with the most stacked pairs. The Make Jump Tree phase is the most computation-intensive part of the entire program, which inherits the core spirit of the Crumple algorithm. It demands time for recursion implementations and disk space to store the solution structures. Thus, any reduction in the input for the Make Jump Tree phase will reduce computational time.

The bundling step bypasses all recursion branches which are guaranteed to resemble the representative structure and therefore saves significant time and space. For two helices to be regarded as similar, we proposed this criterion: the distance from 5′ end to 3′ end must not exceed the length of 4 L + 2 hp – 1, where L is the minimum helix length and hp. is the minimum hairpin loop length, both of which are determined by experimental considerations. There are two reasons for defining similar helices in this manner:

a) From a computational perspective, 4 L + 2 hp. is the minimum length to fit in two adjacent helices. Performing similar helices reduction for 4 L + 2 hp. size or larger allows the possibility of treating *two* adjacent helices “similar” with *one* helix inside the same interval, which is apparently incorrect. The 4 L +2 hp. −1 limit does not allow formation of a multibranch loop in the interval.

b) The wider the span of the interval, the less similar the largest and smallest helices within the inside the same interval will be. Therefore, there must be a fine line somewhere as the cutoff.

For a given interval of size 4 L + 2 hp. – 1 or less, all helices which fit in that sequence space are called similar and are grouped in the same bundle. All helices of the same bundle can be replaced by a representative helix. This representative is selected from the bundle, based on first the helix span and secondly on the degree of saturation, ie the representative helix has the most possible base pairs.

Through this reduction, the total number of solution structures will be greatly reduced and therefore more manageable and analyzable. In addition, a reference list will be provided to contain all possible alternative helices of the same bundle. Note that it is possible for a small size helix to belong to more than one bundle, since bundles can overlap each other, and therefore it is possible that the overlap length is large enough to fit in a small helix. The bundle list implementation is optional. Its pseudo code is included in supporting materials and shows a time complexity of O(m^2^) where m is the number of components.

#### Make jump tree

This phase is the most resource-intensive part of the entire program. All the previous steps have been optimized to improve the efficiency of Make Jump Tree. The core implementation of Make Jump Tree inherits the spirit of the Crumple algorithm. Therefore, it has exponential time complexity. Fortunately, with the effective incorporation of experimental constraints, the exponential coefficient can be scaled down and allow computations on longer RNA sequences.

In the beginning of the function, the interval is examined (Refer to the pseudocode in supporting information). If there is no interval on queue and if it is at the root of recursion (recursion level 0), the entire RNA sequence length is regarded as the interval. For a given interval, components from the component list will be checked one at a time to fit into the interval. If it fits, this component will be inserted into the current structure either as a new helix or to replace a certain old helix. Two new intervals will also be made inside and behind the new helix. The bound of the new inside interval is the inner edges of the new helix. The boundary of the new behind interval is the 3′ end outer edge of the new helix and the 3′ end inner edge of the helix containing the new helix. Then the make_recursion function call is made, and another recursion round begins.

The theoretical time complexity is O(nm) for the worst case where n is the length of the sequence and m is the number of components or bundles. In practice, most of the RNA sequences are rather well-randomized, and therefore the average time complexity is close to O(asqrt(m)), where 1 < a < 3, depending on the experimental constraints and m is the number of components.

The supporting functions that ensure completeness and no duplication of structures add complexity to the code. The descriptions of Restore_Interval and Duplication Prevention, which are part of the Make Jump Tree step, are described next.

#### Restore interval

**Fig. 3:**
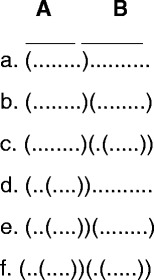
Restore Interval. All secondary structures a-f are valid when single base pairs are allowed. Structures e and f are examples when the behind interval (B) should be conditionally retained. For example, if the B interval was not conditionally retained after evaluating structure b, then structure e would not be generated

For most of the time, whenever an interval is examined, it should be discarded to prevent duplication. However, in some cases, the behind intervals should be conditionally kept after examination and reused later. Fig. 3 illustrates its importance. Solution structures a~f are all correct and necessary, but without the restore interval mechanism, solution structures e. and f. will be missing, because the behind interval (B section) will be examined only once and then discarded.

#### Duplication prevention

The duplication prevention step includes the 2 L – 1 & qLr rules, where L is the minimum length of helix, q is the quotient, and r is the remainder. The first rule states that for a given minimum length of helix, in the case where the minimum helix size and larger are generated, the upper limit of expansion is 2 L-1 in order to prevent duplication because any component of the length 2 L can be replaced by two helices of length L. The second rule is the variant of the general linear equation a = q*d + r, where a, q, d and r are all natural numbers; a is any random number; d is divider; q is quotient; and r is remainder. The qLr rule states that for a helix of any length, a, there must be a way to build it with the helix length of L, L + 1, L + 2... 2 L-2, 2 L-1 (note the 2 L-1 rule limit) without duplication or deletion of structures in the output. So the expression is a = q * L + k, or more precisely, a = (q – 1) * L + (L + k), where k is 0, 1, 2, …, L-1 (note k is remainder).

**Fig. 4:**
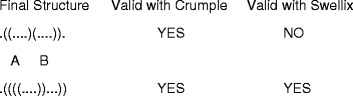
Acceptable Base Pairs in Crumple and Swellix. The top structure highlights an example when Swellix would not allow a structure if a minimum helix length of two is a constraint. This constraint effectively removes single, isolated, unstacked pairs, also known as “lonely pairs”

Note that in Swellix, helix length constraints like a minimum helix length of 2, which effectively eliminates any single, isolated, unstacked pairs, will produce a smaller output set than an algorithm such as Crumple. For Swellix, these length constraints are enforced in the first step when it is analyzing the sequence for possible components, ie individual helices. With Crumple, however, these length constraints will not be enforced until a full possible structure has been assembled with base pairs. At this point, the program looks for violations. For example, if a minimum helix length of 2 is applied, the output of Crumple and Swellix may differ as shown in Fig. 4. With Swellix, helices A and B in the first structure never make it past the initial step by being added to the set of components for consideration because they are not at least 2 base pairs long. Thus, Swellix efficiently eliminates single, isolated, unstacked pairs that do not occur in natural RNA structures. In addition, this adds to the reduction of the size of the component set that will be considered combinatorically.

#### Parallelization

Bundling and the recursive “jump tree” algorithm do not scale well with input size. The bundling process has the effect of, given an *n*-nucleotide sequence, processing *n*-many subsequences before beginning the standard recursion. This negative performance impact is magnified by factors such as sequence length and runtime options like minimum helix length constraints. Bundling was identified as a parallelization candidate due to the data-independence of those *n*-many subsequences to be processed. Bundling and the recursive jump tree are similar in that they both have high potential for parallelization. They are unique in the type of parallelization necessary for efficient scaling.

The nature of the bundling algorithm can be abstracted as such: for *n* nucleotides in a sequence, there will be *n* units of work in the bundling step. Each unit of work consists of running a slightly modified version of Crumple on some substring of the RNA input sequence. The results from these computations are consolidated and used to improve the speed of Swellix’s recursion. For each of these substrings from the input sequence, only the data in one substring is required for its respective computation. Since each “unit” of work is completely separate from the others, we chose to parallelize the bundling stage by dividing the “units” equally among our pool of MPI (Message Passing Interface) processing elements. The end result is akin to an OpenMP parallel for loop: each MPI core uses its own rank, the world size, and the length of the RNA sequence to calculate at what index to start, and how many iterations of the loop to process.

For Make Jump Tree, parallelization is more complex. The same strategy used for bundling could be used here, but the recursion is much more imbalanced than bundling. In an effort to achieve the most balanced compute load as possible, we chose to implement a dynamic load balancing algorithm for the recursive parallelization. This parallelization relies on each node having a copy of the component list. Then each node creates an array of pointers to each component for rapid access instead of iterating through a linked list.

Phase 1 of the parallelization strategy can be described as a coarse master/worker paradigm. The master node begins a loop from 0 to *n* (*n* = # components) in which it probes the MPI network for work requests from idle nodes. When it detects such a request, the master sends its current loop index to the node, indicating which index in the component array the worker node should use for a new recursion. Immediately after the master sends the *n* ^*th*^ index, it sends another message to all nodes in the MPI pool indicating that Phase 1 is complete. During phase 1, the worker nodes begin their procedure by sending a work request message to the master node. Then, they pause execution to wait for some response. If they receive a response with an array index, then the worker accesses the component array at that index and begins a recursion using the same function call that serial Swellix uses normally. Once a recursion is complete, the worker again requests work and waits for a response. Upon receipt of the phase 1 completion message, a worker node simply continues to the data consolidation stage where it will send the information it generated back to the master node for display.

During phase 2, a version of Dijkstra’s token rings that were applied to parallelization of the Crumple algorithm were used again [21, 27, 28]. If a node is busy in its recursion tree, then it regularly probes for a “work requested” message. If the node detects a work request message, then it breaks off a branch of its tree and sends the information required to continue down that branch over to the node who requested work. If a node detects a request for work and isn’t ready to send work, the node should pass the message around the “ring” of processors. After the detection that all nodes are finished, a kill signal is passed around indicating that the processor nodes can finish the recursive portion and display the results.

### Computational resources

Early- to mid-stage development was done on University of Oklahoma resources including both the Boomer and Schooner Linux cluster supercomputers. For the Boomer computations, the compute nodes used contained the following hardware: 2 Intel Xeon E5–2650 “Sandy Bridge” 8-core 2.0 GHz processors with 32 GB of RAM. The compute nodes that we used on Schooner have the following specifications: 2 Intel Xeon E5–2650v3 “Haswell” 10-core 2.3 GHz processors with 32 GB of RAM.

Late-stage development took place almost exclusively on the Blue Waters supercomputer through the Blue Waters Project and Shodor Education Foundation’s Blue Waters Student Internship Program. The typical compute node used on Blue Waters was the XE6 node: 2 AMD 6276 Interlagos 16-core (“integer” core) 2.45 GHz processors with 64 GB of RAM.

## Results and discussion

### Swellix performance

**Fig. 5:**
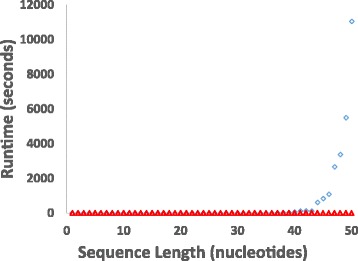
Comparison of Crumple and Swellix Times for RNA of Different Sequence Lengths. The graph of run-time versus sequence length shows how the run time for Crumple grows exponentially after 35 nucleotides. Crumple is run with no lonely pairs (blue diamonds) and Swellix is run with a minimum helix length of two (red squares). Each run time is the average of 10 repetitions of the calculation

Swellix enables longer RNA sequences to be computed and analyzed. Swellix with a minimum helix length of one is nearly identical to Crumple. The performance analysis in Fig. 5 shows a comparison between Swellix with minimum helix length of 2 and Crumple with “no lonely pairs”, ie no isolated, single, unstacked pairs. The test consists of 10 trials, each with 50 unique, randomly generated sequences of length 1–50. Each sequence in a trial was 1 nucleotide longer than the previous. This produced a total of 10 data points for each sequence length 1–50, and these data points were averaged for a resulting value of runtime versus sequence length. With out bundling, Swellix performs nearly the same as Crumple from 1 to 35 nucleotides. At this point, Swellix and its additional computational procedures and improvements in efficient memory use greatly speed up runtime while Crumple begins to slow down with apparently exponential trend. The completeness and accuracy of Swellix was checked by comparing the Swellix output for minimum helix length of 1 with Crumple output. Table 1 provides additional benchmarks for run times for biological sequences of different lengths using the Swellix program on the Schooner computer. As observed in the column of run times without bundling in Table 1, Swellix runtimes with a minimum helix length of 2 do not begin to grow exponentially in run time until approximately 70 nucleotides.

**Table 1:** Swellix Run Times for RNA Sequences

Sequence	Nucleotides	Components	Components w/ bundling	Bundles	Structures	w/ bundling	Runtime (s)	Runtime (s) w/ bundling	Bundling Time Efficiency (%)
14mer	14	12	8	8	15	8	0.2	0.29	
28mer	28	53	40	28	833	374	0.2	0.4	
42mer	42	130	108	48	67,014	25,862	0.35	0.58	
MicA	50	221	193	71	1,127,719	391,111	3.27	1.7	48.0
tRNA asp 7	71	578	526	141	14,676,607,199	3,586,825,719	51,708.28	12,627.35	75.6
tRNA RG1660	74	471	436	98	2,098,681,265	658,285,383	7140.63	2216.78	69.0
tRNA 1EHZ	76	533	495	107	3,820,164,477	1,327,023,534	13,577.62	4685.58	65.5

Swellix computations were run with and without the bundling option on the Schooner computer and a minimum helix length of 2. The reduction in the number of components, the number of structures, and the runtimes with the bundling option are highlighted in blue. Bundling time efficiency is calculated as the difference in runtimes with and without bundling divided by the runtime without bundling. For the very short sequences with less than 1 s runtime, the time to perform the bundling step increases the overall runtime. The 14mer sequence is 5′5’GCUCUAAAAGAGAG 3′. The 28mer and 42mer are concatamers of the 14mer sequence. The MicA sequence is a domain of a bacterial noncoding RNA [40]. The three tRNA sequences are tRNA Asp 7 from *Homo sapiens* [41], tRNA Ala from *Shigella sonnei* [42], and tRNA Phe from *Sacchromyces cerevisiae* (PDB# 1EHZ) [43]

The abstract helix representation in bundling further improves the ability of Swellix to compute longer sequences in a reasonable time. In order to test whether bundling of similar helices was the prime contributing factor to Swellix’s performance compared to Crumple, sequences of increasing lengths were used: 14, 28, 42, 50, 71, 74, and finally 76 nucleotides. The first three sequences are artificial sequences used for general testing and development. The “14-mer” is 5’GCUCUAAAAGAGAG3’ and was designed to produce a set of structures that contain an example of each possible kind of soft constraint for DMS-modified adenines. The 28- and 42-mer are 2 and 3 concatamers of the 14-mer sequence. The longest sequence is a tRNA sequence, yeast phenylalanine tRNA (crystal structure PDB #1EHZ). As shown in Table 1, the Bundling feature reduces the size of the input which gets passed to the recursive portion of Swellix. Note that the bundled structures can be “unbundled” and thus this computational improvement doesn’t come at the cost of solution completeness. This input size reduction inherently also reduces output size, and it follows that the runtime would also be decreased.

As a computational exercise several tRNA, pRNA, and group I intron sequences were folded on the Blue Waters computer using the parallelized bundling option. The minimum helix length was varied from 2 to 10. Table 2 shows the results of this exercise for the tRNA 1EHZ sequence. This exercise revealed a “sweet spot” in the bundling computations. For very short sequences, the bundling computation added time. For very long minimum helix lengths, the length of the sequence to be analyzed according to the 4 L + 2 hp – 1 rule and the computations to create the bundles add increasing amounts of time. The best balance for the benefits of bundling occur with a minimum helix length of 4 or 5. Table 3 shows the results of folding sequences up to 247 nucleotides with a minimum helix length of 5. The fast run times for longer sequences demonstrate that complete enumeration is possible given sufficient experimental constraints. Table 4 shows the parallelization of tRNA and three HERV RNA sequences up to 418 nucleotides with minimum helix lengths ranging from 2 to 6 on the Blue Waters computer. A comparison of the serial and parallel run times provides an estimate of the parallelization efficiency, which has complex dependencies on sequence length, number of bundled components, and minimum helix length. The highest ratios of sequential to parallel run times occur in cases with a large number of bundled components and a short minimum helix length. Although a minimum helix length of 2 is practical and reasonable assumption for biological sequences without further experimental data, additional experimental constraints can be generated from in vivo chemical probing of paired nucleotides, ie the PARIS (psoralen analysis of RNA interactions and structures) method [2], or cryoelectron microscopy [26, 29]. Both the PARIS method and cryoelectron microscopy are revealing an increasing number of RNA with multiple conformations and multiple folds. Thus, Swellix will be a complementary tool to analyze RNA sequences with multiple folds .

**Table 2:** Effect of Helix Constraints on Bundling of a 76-mer tRNA

Minimum Helix Length	Components	Bundles	Structures	Runtime (s)
2	495	107	1,327,023,534	6875.18
3	215	56	563,026	8.06
4	91	28	8292	5.5
5	36	11	314	21.02
6	13	5	33	293.08

Swellix computations were run with the parallelized bundling option on the Blue Waters computer. The sequence is the 1EHZ tRNA

**Table 3:** Swellix Computations of Longer RNA Sequences

Sequence	Minimum Helix Length	Nucleotides	Components	Bundles	Structures	Runtime (s)
MicA	5	50	9	9	22	27.45
tRNA asp 7	5	71	31	14	271	43.61
tRNA RG1600	5	74	22	12	254	14.51
tRNA 1EHZ	5	76	36	11	314	21.02
GA1 pRNA	5	161	154	26	417,535	29.43
SF5 pRNA	5	167	206	31	2,679,059	54.09
M2 pRNA	5	171	185	31	2,988,931	60.79
phi29 pRNA	5	174	270	57	47,596,862	200.28
Azoarcus gr. I intron	5	197	173	32	3,020,337	30.11
Tetrahymena gr. I intron	5	247	247	35	105,405,879	285.49

Swellix computations were run with the parallelized bundling option on the Blue Waters computer. The sequences are those in Table 1 as well as 4 prohead RNA sequences [44] and 2 group I intron sequences [45]

**Table 4:** Comparison of Serial and Parallel Runtimes for Longer RNA Sequences

Sequence	Minimum Helix Length	Bundled Structures	Parallel Runtime (s)	Serial Runtime (s)
tRNA-1EHZ	2	1,327,023,534	456.28	11,117.55
tRNA-1EHZ	3	563,026	3.25	7.61
tRNA-1EHZ	4	8292	4.71	10.24
tRNA-1EHZ	5	314	45.32	137.53
tRNA-1EHZ	6	33	718.27	2498.52
HERV 141	3	12,518,055,094	5550.62	112,162.24
HERV 141	4	1,463,580	8.06	22.24
HERV 141	5	3401	41.97	130.4
HERV 141	6	43	663.51	2303.08
HERV 244	5	195,971,275	256.02	2229
HERV 244	6	59,909	2116.68	7239.21
HERV 418	6	7,514,046,365	10,040.77	97,296.14

Swellix computations were run with the parallelized bundling option on the Blue Waters computer. Each sequence was given the same number of cores as nucleotides in the sequence, ie tRNA-1EHZ has 76 nucleotides and cores. There are three HERV RNA sequences of different lengths: 141, 244, and 418 nucleotides [46]. HERV141 is the shortest known HERV RNA fragment, and HERV418 is a self-folding domain that binds the Rev. protein [47]

### Swellix analysis of the effects of naturally chemically modified nucleotides in tRNA

One of the roles of modified nucleotides in tRNA is to reduce conformational space and prevent misfolding [30]. Swellix was used to quantify the reduction in conformational space for individual modifications and the collective effect of modifications in 17 tRNA sequences. 17 sequences (Additional file 1: Table S1) were selected from the tRNA database [31]. Each sequence contains 76 nucleotides and varying numbers of total modified nucleotides that interfere with Watson-Crick base pairing. For example, given a sequence with a total of 9 chemically modified nucleotides that prevented Watson-Crick base pairing, 10 variants of the sequence were created. The first variant would instruct Swellix to fold the sequence without constraints as if there were no modified nucleotides. The next would enforce the first modified nucleotide not to pair, and so on until the final variant enforced constraints on all the chemically modified nucleotides. The order of constraint enforcement depended on the distance from the 5′ end. The first modification to be enforced was the 5′-most modification and the last was the one closest to the 3′ end. With these variants created, we ran them all in serial Swellix with bundling on and with a minimum helix length of 2 on the Boomer computer. The longest computation took ~11.12 h, with the average runtime being ~1.18 h. In parallelized Swellix, tRNA computations run in less than a minute on the Blue Waters computer (Tables 3 and 4).

**Fig. 6:**
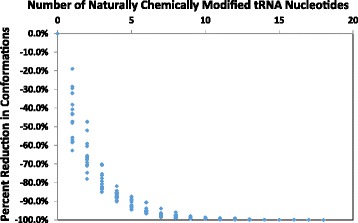
Effect of tRNA Modifications on Conformational Space. This graph shows the percentage reduction in the number of conformations as a function of the number of constraints applied to naturally chemically modified nucleotides for 17 tRNA sequences. The percentage reduction in folding space is calculated from the total number of possible structures computed for the sequence with and without constraining naturally chemically modified nucleotides to be single stranded. All tRNA sequences were computed with bundling and a minimum helix length of 2 on the Boomer computer

Fig. 6 demonstrates the relationship between reductions in output size versus the number of enforced constraints for naturally chemically modified nucleotides. Notably, the trend isn’t linear. Subsequent enforced modifications don’t necessarily cause the same amount of folding reduction as their predecessors. Overall, the most significant reduction in number of possible folds occurs with the first constraint for modified nucleotides. The quantification of the reduction in the number of possible folds enables an estimate of the entropic benefits of naturally chemically modified nucleotides. The estimate of the entropic benefit can be calculated using the Second Law of Thermodynamics (Eq. 1)

$$ \mathrm{S}=\mathrm{k}\ \ln\ \mathrm{W} $$

where S is entropy, k is Boltzman’s constant, and W is the number of possible RNA folds. Thus, comparing the number of possible tRNA folds for 16 unmodified sequences versus the fully modified sequences, the average entropic difference in the tRNA folding reaction is −3.9 ± 0.4 J/molK per modified nucleotide. The average entropy reduction for the first modified nucleotide is −4.9 ± 1.9 J/molK. The maximum entropic benefit for the most modified sequence, a tryptophan tRNA from *Triticum aestivum*, is −73.2 J/molK. Thus, our computations confirm and quantify one role for naturally modified nucleotides in reducing conformational space for RNA folding.

The above example illustrates how chemical modifications in natural RNA limit folding space. One further application of the Swellix program in the field of synthetic biology would be to estimate the optimal location in a sequence to restrict folding with a nucleotide unable to pair. When designing RNA sequences, incorporation of chemically modified nucleotides are one method to direct folding and restrict folding space in particular ways or regions of the sequence. Thus Swellix may also facilitate rational design of sequences with engineered folds.

### Swellix analysis of protein and drug binding motifs in HERV RNA

**Fig. 7:**
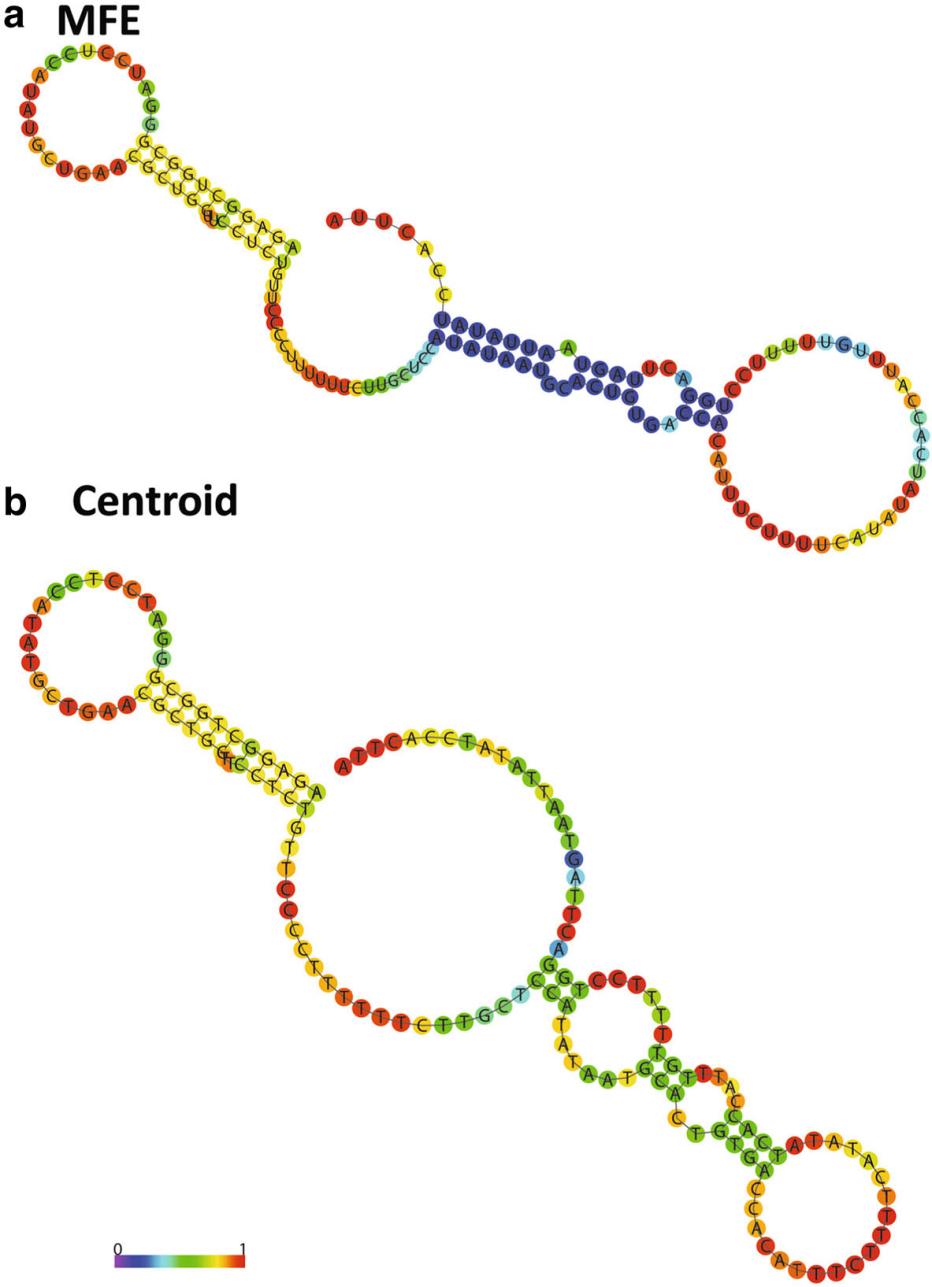
HERV Minimum Free Energy and Centroid Structures. The minimum free energy structure (MFE) and centroid structure for the 141-nucleotide HERV sequence (Genbank # AY944072.1)are shown. Base pairs are colored according to base pair probabilities computed with thermodynamic parameters using the Vienna Program [38]. The same sequence folded using the RNAStructure program generates an MFE structure and 6 additional suboptimal structures, none of which contain the motifs in Table 5

The shortest Human Endogenous Retroviral RNA (HERV) sequence is 141 nucleotides (Genbank # AY944072.1). Folding this HERV RNA using the parallelized Swellix on the Blue Waters computer generated 643 components and 12,518,055,094 bundled structures in an average time of 23,951 s. Swellix does not save all the generated structures, but a motif search can be completed for each structure before it is discarded. A search for known RNA motifs [32–37] from HIV and Hepatitis viral RNA yielded the results shown in Table 5. Although these protein and drug binding motifs do not appear in the minimum free energy structure, suboptimal structures, or centroid structures predicted by Vienna [38] or RNAStructure [39] programs (Fig. 7), the motif occurrence can be as high as 4.5%. Thus, Swellix can search the entire conformational ensemble to identify motifs that might otherwise be overlooked. The thermodynamic-based predictions do not yield any structures with high base pair probabilities, and the MFE structure occurrence is only 1.17% of the ensemble. This HERV sequence does not have one thermodynamically dominant predicted structure, and thus Swellix is an alternative approach to RNA folding that can provide new insights, such as revealing the presence of motifs that bind therapeutics.

**Table 5:** Motif Searches in Swellix Analysis of HERV RNA

Motif			% occurrence
GNRA tetraloop	WWWGNRAWWW	(((....)))	0.00
Hepatitis C 1	WWGAACUACWW_WWGCWW	(((.....(((_))))))	4.41
Hepatitis C 2	WWUACCCACCWW_WWGAGWW	(((......(((_))).)))	2.71
HIV TAR	WWAUCUGWW_WWCUWW	(((...(((_))))))	4.58
HIV and 7SK	WWUCUUWW_WWARWW	(((..(((_))))))	0.73
HIV RRE 1	WWUGGAAWW_WWUGGGAGWW	(((...(((_)))....)))	2.80
HIV RRE 2	WWGGGCWW_WWGGUACWW	(((..(((_)))...)))	4.58

A 141-nucleotide Human Endogenous Retroviral (HERV) RNA sequence (Genbank # AY944072.1)was folded using a phase 1 parallelized Swellix on the Blue Waters XE nodes with 16 cores per node. The computation generated 643 components and 12,518,055,094 bundled structures in an average runtime of 23,951 s. The minimum helix length constraint is 3. The motifs that were counted are RNA loops, some of which bind small molecule drugs in HIV Trans Activating Response (TAR) or Rev. Responsive Elements (RRE), 7SK RNA, or Hepatitis C RNA [32–37]. For the motif sequence, W indicates a Watson-Crick pair; N indicates any nucleotide; and R indicates a purine. In the sequence and dot and parentheses notation, an underscore indicates any variable number of intervening nucleotides. The percent occurence is the number of times the motif was counted during the computation divided by the total number of structures generated

## Conclusion

Swellix effectively combines helix abstraction with a combinatorial approach to RNA structure determination in order to efficiently compute all possible non-pseudoknotted structures for an RNA sequence. Swellix can analyze an RNA up to 418 nucleotides on the Blue Waters Supercomputer, and thus demonstrates that computer time and nodes are the only limits to a combinatorial approach to the RNA folding problem. The current capabilities of modern supercomputers and efficient tools such as Swellix make combinatorially complete searches of RNA conformational space a realistic option and dispel the myth of impossible RNA computations. Swellix is capable of incorporating unique constraints, such as the minimum number and length of helices, from crystallography or cryoelectron microscopy experiments. The possible biological applications for Swellix are demonstrated by computing the entropic benefits of reducing conformational space with modified nucleotides in tRNA folding and motif abundance in HERV RNA folding. Swellix reveals protein and drug binding motifs that occur in the entire ensemble but do not occur in the predicted minimum free energy or centroid structures. Thus, Swellix provides an alternative approach to RNA structure analysis when the assumptions of free energy minimization do not apply or when multiple conformations are present.

## Additional file

Additional file 1: Supporting Information. Supporting Information includes the pseudocode and readme file for the Swellix program and **Table S1**, a table of tRNA sequences and modifications. (PDF 86 kb)

## Abbreviations

HERV: Human endogenous retroviral RNA
MPI: Message passing interface
