# Supplementary material for: Swellix: a computational tool to explore RNA conformational space
Source: BMC Bioinformatics. 2017 Nov 21;18:504. doi: 10.1186/s12859-017-1910-7 (PMC5697422; doi:10.1186/s12859-017-1910-7)
Supplement: Additional file 1: — Supporting Information. Supporting Information includes the pseudocode and readme file for the Swellix program and Table S1, a table of tRNA sequences and modifications. (PDF 86 kb) [file 12859_2017_1910_MOESM1_ESM.pdf]

Supporting Information for
Swellix: a Computational Tool to Explore RNA Conformational Space

Nathan Sloat[†], Jui-Wen Liu[†], Susan J. Schroeder*

[†] These authors contributed equally to this work.

*Corresponding author

Shortened title for running head: Swellix Explores RNA Conformational Space

Keywords: RNA structure prediction, RNA ensembles, conformational space, RNA motif search

Pseudocode

Pair Table

```
for i in RNA sequence 5' pairing range:
    for j in RNA sequence 3' pairing range:
        if nucleotide i can pair with nucleotide j:

            write 1 in corresponding table cell

        else:

            write 0 in corresponding table cell
```

Component List

```
for k_1 in RNA sequence 5' pairing range:
    for k_2 in RNA sequence 3' pairing range:
        for k_3 of various helix sizes:
            for (i, j) as 5' and 3' nucleotide pair along the candidate component:
                if (i, j) can be paired:
                    mark a parenthesis pair
                else:
                    mark a mismatch
            if component is qualified as prescribed perfect or mismatch helix:
                insert new component into the component list
```

Interval Lookup Table

```
for i in RNA sequence 5' pairing range:
    for j in RNA sequence 3' pairing range:

      for component of component type i:
            if component fit in (i, j):
                update lower/upper bounds of cell (i, j) of interval look-up table
```

Bundle List

```
make_recursion_inside_helix:

    for component in list:

        if component fit in:

            check_qualifications

            save_qualified_bundle

            make_recursion_inside_helix
```

Recursive Jump Tree

```
make_recursion:
    for interval on queue:
        for component in list:
            if component fit in interval:
                check_qualifications
                update_sequence
                save_qualified_structure
                make_interval_inside_component_to_push_on_queue
                make_interval_behind_component_to_push_on_queue
                make_recursion
```

Table S1. Table of tRNA sequences and modifications

tRNA Sequence	# Naturally Modified Nucleotides
Nanoarchaeum equitans Gln	0
Mycoplasma capricolum Ile	3
Mycoplasma capricolum Val	4
Escherichia coli Ala	5
Bacillus subtilis Ala	6
Escherichia coli Thr	7
Bacillus subtilis Lys	8
Homo sapiens His	9
Drosophila melanogaster His	10
Haloferax volcanii Pro	11
Bombyx mori Ala	12
Bos taurus Arg	13
Lupinus luteus Phe	14
Mus musculus Arg	15
Avian myeloblastosis virus Met	16
Bos taurus Phe	17
Triticum aestivum Trp	18

swellix
Here, we'll cover some of the basics of computing with Swellix.

First: Compiling Dependencies

THE VIENNA PACKAGE:
Swellix uses some utilities provided in the ViennaRNA package for computing thermodynamics and RNA distance in structures. Swellix calculates thermodynamics only after Swellix has computed structures. Free energy calculations are not required for Swellix computations but can be useful when comparing Swellix output to the output from other RNA prediction software. When you download Swellix, you should get a copy of Vienna: `ViennaRNA-2.2.5.tar`
To configure Swellix in a simple way, you'll first need to decompress the ViennaRNA tarball in the same directory as
the Swellix Makefile:

```
tar -xf ViennaRNA-2.2.5.tar
```

You should now have a new directory called "ViennaRNA-2.2.5." The next step is to compile the ViennaRNA
library:

```
make vienna
```

This should populate the `viennabuild` directory with the resources that Swellix will use during computation. Inside of this `viennabuild` directory, there will be an existing thermodynamic parameter file called `rna_turner2004.par`. This file
informs the calculations that are performed at runtime to determine the free energy of a secondary structure. If you have
a different or updated parameter file that you'd like to use then you can simply replace `rna_turner2004.par` with your
file. For this file to be properly used, you must also make a change to the Swellix Makefile. In the Makefile, there is a
variable `PARAMFILE`. In this variable, you must change `rna_turner2004.par` to the name of the file you want to use.

Second: Compiling Swellix

The Swellix Makefile has gathered multiple options for compilation. The ones of most use are:

SERIAL:
Compilation using
```
make serial
```

will provide the serial version of Swellix with the most basic needed output. The program
will output the RNA sequence it was given, and the number of structures computed. You may provide Swellix the commandline
flag `-d` with a value of 2 to print each structure out in dot & parenthesis format to the terminal as it is computed.

MPI:
Using
```
make mpi
```
will produce a result similar to `make serial` except the code will be compiled to run using the parallel
version of the algorithm. Once compiled, you should be able to run Swellix with `mpirun` or the like.

NOTE
The parallel code in Swellix was developed using OpenMPI, so problems could arise if using some other implementation of MPI.

DISP (Display):
Compiling with
```
make disp
```
instructs Swellix to provide various levels of more detailed output. These levels depend on the
`-d` flag as detailed at the bottom of this file in the table of commandline options.
Level 1 is the same as the default output from using `make serial` above except with more information relating to the data
structures that were used in the algorithm. This information is mainly the size of some lists.
Exceeding Level 2 results in output which is really only useful for debugging or further development.

NOTE
If you do choose to use the `-d` option for more detailed output, be wary of the size of your sequence and possible size of
output. You can easily generate very large files from the output of structures alone. This is not to mention the debugging
text if you have the display level set high enough. It all comes down to your imposed constraints.

Third: Input Format

Swellix RNA sequence input must be formatted properly before being run in the program.

GUIDELINES/RULES:
The first rule is the simplest, and we'll call it the 'One Line Rule'. Any input sequence should occupy only one line.

e.g.
Say you have some arbitrary sequence `GCUCUAAAAGAGAG`. You shouldn't create an input file with your sequence formatted on two lines like this:
```
GCUCUAA
AAGAGAG
```
This is because it contains a new line indicator after the first 7 nucleotides. Swellix can't tell if the two lines are meant to be the same sequence or if you're trying to give it some kind of multi-sequence input, which the program doesn't currently handle. The result of this input would be Swellix running with only the first line as its input.

The second rule deals with what characters are used to represent the sequence. Swellix only knows how to handle sequences which consist of A, C, G, and U representing the nucleotides. If there are spaces, place holders, nucleotides with chemical modifications, etc., Swellix will not properly handle the sequence. The output from the program will be either incorrect or, even worse, the program will crash. So, any special characters need to be either removed or properly converted back to their corresponding A, C, G, or U.

e.g.
Imagine that you have a sequence to run which contains some place-holding characters. For example, let the sequence look like `GCUCU--AAAAGA---GAG`.
Since Swellix doesn't know what to do with these hyphens, you must first strip them from the sequence and then adhere to the previous one line rule. So, when sending this sequence to Swellix it should look exactly like the previous example: `GCUCUAAAAGAGAG`.

e.g.
Now consider the case where there is some number of modified nucleotides which constrain the folding of the RNA. Let the sequence be `GCUCU"AAAKAGAG`, where `"` represents the 1-methyladenosine modification and `K` represents the

1-methylguanosine modification. (these modifications are arbitrarily chosen for the example)
We've stated that Swellix can't handle these characters properly on its own, so we need to first convert them to their unmodified characters. So the sequence will once again look like `GCUCUAAAAGAGAG` to Swellix.

Fourth: Providing Input to Swellix

Swellix can accept input in two ways: standard input, and an input file specified with the `-i` flag. In addition, you can specify most folding constraints via the commandline. For the others, you must provide them in you configuration file.

STANDARD INPUT:
To use standard input, simply pipe a sequence to Swellix like so:
```
echo "GCUCUAAAAGAGAG" | /path/to/swellix/swellix.exe [desired constraints]
```
where the desired constraints are some optional combination of the flags defined at the bottom of this file.

FILE INPUT:
There are two flavors of reading input from a file. In one, only the first line of the file, which should be the sequence, is read into the program. In the other, you can instruct Swellix to continue reading through the file to look for any defined constraints. For the second case, we'll refer to that file as a configuration file since it is providing Swellix with more information than just the sequence.

In general, to use a plain input file you need the `-i` flag and the path to the file.
```
/path/to/swellix/swellix.exe -i sequence.txt [optional command-line args]
```

However, if you would like to provide a configuration file with certain folding constraints, you will also need to include the `-k` flag. You will still use the `-i` flag and the path to your config file.
```
/path/to/swellix/swellix.exe -k -i config.swlx [optional args/constraints not in config file]
```

ABOUT THE CONFIGURATION FILE:

There are many constraints that can be imposed just by command line arguments. The advantage of providing input via a config file is that you can specify constraints such as individual nucleotide pairing restrictions. For example, you can tell Swellix that any particular nucleotide absolutely must pair to form a valid structure. The constraints provided must be in a strict format for the time being. The specific formatting rules and an example of a properly written config file is provided. It is called `configTutorial.swlx`. In this file, we use the same sequence as above but illustrate how to specify constraints.

NOTE
If the case arises where you have provided input via both standard input and an input file, the sequence defined by the input file will override the sequence provided by standard input.

Swellix Configuration Tutorial

This document is for specifying and demonstrating the format restrictions on the constraints within a Swellix configuration file. For this example, we'll be using the same 14 nucleotide sample sequence which was our focus in the README file. Specifically, we'll use the version of the sequence which had chemically modified nuceleotides to restrict pairing possibilities.

As a refresher, our sequence was 'GCUCU"AAAKAGAG'. The '"' and the 'K' are our modified nucleotides, and the first step is always to obtain the "unmodified" sequence. This just means replacing the symbols for the modified nucleotides with the corresponding symbols for the unmodified nucleotides.
So, the sequence that we want to provide to Swellix looks like this: 'GCUCUAAAAGAGAG'.

Next, we need to translate the pairing constraints (if any) to Swellix.

For now, the syntax for pairing constraints consist of three key phrases: "S1 PAIRING", "V1 PAIRING", and "COVARIANCE".

S1 PAIRING is used to denote a list of indices which identify nuceleotides which MUST NOT pair.
V1 PAIRING is used to denote the opposite of S1: indices of nucleotides which MUST pair.
And COVARIANCE constraints list ordered pairs indicating pairs of nucleotides which must pair with each other.

For our example, we are assuming that the chemical modifications prohibit pairing of the respective nucleotides, so we will create a config file using the S1 PAIRING indicator.

The first step is always the same as for a normal sequence input file: enter the sequence on the first line of the file.

So, the first line will be:

```
GCUCUAAAAGAGAG
```

Next, on a new line below the sequence, specify the type of pairing constraint using square brackets and the necessary key word/phrase. In our case, it is [S1 PAIRING]. Then, for each nucleotide to be constrained, enter the index surrounded by parentheses. So, the pairing constraint section for our test sequence will look like this:

```
[S1 PAIRING]
( 6 )
( 10 )
```

A few notes: the current implementation in Swellix requires there be no spaces between the brackets and the keyword. Inversely, it is strictly required that there be spaces between the parentheses and the index. This will be made more flexible in a future revision.

So, our complete config file would look like this:

```
GCUCUAAAAGAGAG
[S1 PAIRING]
( 6 )
( 10 )
```

This is a simple example, and you could easily have more complicated constraint variations. If you want to add constraints of more than one type to your sequence, you can separate the different types of constraints by a line of underscores.

For example, for some arbitrary sequence that had constraints of all three types, you could have something looking like this:

```
[V1 PAIRING]
( 5 )
( 24 )
( 340 )
___________
[S1 PAIRING]
( 2 )
( 56 )
( 100 )
___________
[COVARIANCE]
( 20 , 79 )
( 134 , 300 )
```

The number of underscores is not strict. Only one is required on the line separating the constraints. However, you can write it as shown to make it more pleasing to human eyes.

If you enter your constraints and receive output that is obviously erroneous or the program crashes, make sure that the formatting is correct first. If the error persists and you don't want to troubleshoot it yourself, contact us with the bug description and we'll work to fix it and update the code.
